# Burden of congenital rubella syndrome (CRS) in India based on data from cross-sectional serosurveys, 2017 and 2019–20

**DOI:** 10.1371/journal.pntd.0009608

**Published:** 2021-07-23

**Authors:** Devika Shanmugasundaram, Shally Awasthi, Bhagirathi Dwibedi, S. Geetha, Manish Jain, Shikha Malik, Bhupeshwari Patel, Himabindu Singh, Shalini Tripathi, Rajlakshmi Viswanathan, Anjoo Agarwal, Rajeswari Bonu, Shuchi Jain, Saubhagya Kumar Jena, J. Priyasree, K Pushpalatha, Syed Ali, Debasis Biswas, Amita Jain, Rahul Narang, Sudha Madhuri, Suji George, Ojas Kaduskar, G. Kiruthika, R. Sabarinathan, Gajanan Sapakal, Nivedita Gupta, Manoj V. Murhekar

**Affiliations:** 1 ICMR–National Institute of Epidemiology, Chennai, Tamil Nadu, India; 2 King George Medical University, Lucknow, Uttar Pradesh, India; 3 All India Institute of Medical Sciences, Bhubaneswar, Odisha, India; 4 Govt Medical College, Thiruvananthapuram, Kerala, India; 5 Mahatma Gandhi Institute of Medical Sciences, Sewagram, Maharashtra, India; 6 All India Institute of Medical Sciences, Bhopal, Madhya Pradesh, India; 7 Nilofer Hospital, Hyderabad, Telangana, India; 8 ICMR–National Institute of Virology, Pune, Maharashtra, India; 9 All India Institute of Medical Sciences, Bibinagar, Telangana; 10 Indian Council of Medical Research, New Delhi, India; Beijing Children’s Hospital, Capital Medical University, CHINA

## Abstract

**Background:**

India has set a goal to eliminate measles and rubella/Congenital Rubella Syndrome (CRS) by 2023. Towards this goal, India conducted nationwide supplementary immunization activity (SIA) with measles-rubella containing vaccine (MRCV) targeting children aged between 9 months to <15 years and established a hospital-based sentinel surveillance for CRS. Reliable data about incidence of CRS is necessary to monitor progress towards the elimination goal.

**Methods:**

We conducted serosurveys in 2019–20 among pregnant women attending antenatal clinics of 6 hospitals, which were also sentinel sites for CRS surveillance, to estimate the prevalence of IgG antibodies against rubella. We systematically sampled 1800 women attending antenatal clinics and tested their sera for IgG antibodies against rubella. We used rubella seroprevalence data from the current survey and the survey conducted in 2017 among antenatal women from another 6 CRS surveillance sites to construct a catalytic models to estimate the incidence and burden of CRS.

**Result:**

The seroprevalence of rubella antibodies was 82.3% (95% CI: 80.4–84.0). Rubella seropositivity did not differ by age group and educational status. Based on the constant and age-dependent force of infection models, we estimated that the annual incidence of CRS in India was 225.58 per 100,000 live births (95% CI: 217.49–232.41) and 65.47 per 100,000 live births (95% CI: 41.60–104.16) respectively. This translated to an estimated 14,520 (95% CI: 9,225–23,100) and 50,028 (95% CI: 48,234–51,543) infants with CRS every year based on age-dependent and constant force of infection models respectively.

**Conclusions:**

Our findings indicated that about one fifth of women in the reproductive age group in India were susceptible for rubella. The estimates of CRS incidence will serve as a baseline to monitor the impact of MRCV SIAs, as well progress towards the elimination goal of rubella/CRS.

## Author summary

Rubella infection during the first trimester of pregnancy can affect fetus, resulting in spontaneous abortion, stillbirth or birth of a baby with a combination of birth defects known as congenital rubella syndrome (CRS). Vaccination with rubella containing vaccine (RCV) is recommended as one of the strategies for eliminating rubella/CRS. The Southeast Asia region has set a target to eliminate rubella/CRS by 2023. Towards this goal, India completed nationwide immunization campaigns using measles-rubella vaccine during 2017–19, targeting children aged 9 months to <15 years. A case-based surveillance for CRS was initiated in five sentinel hospitals (Phase-1) in 2016 and later expanded to additional 6 sites (Phase-2) in 2019, to estimate burden of CRS and monitor its trend. As an adjunct to CRS surveillance, periodic serologic surveys were also planned to monitor the rubella seroprevalence among the pregnant women. A serosurvey conducted in 2017 indicated that 83.4% pregnant women attending antenatal clinics of Phase-1 sentinel hospitals had IgG antibodies against rubella. The second serosurvey conducted during 2019–20 in 6 Phase-2 sites indicated a comparable seroprevalence of 82.3%. Using seroprevalence data from these two serosurveys, we estimated that the annual incidence of CRS in India was 225.58 per 100,000 live births with constant force of infection and 65.47 per 100,000 live births with age-dependent force of infection models. This incidence rates translated to an estimated 14,520 to 50,028 infants with CRS every year. The estimates of CRS incidence will serve as a baseline to monitor the progress towards the elimination goal of rubella/CRS in India.

## Introduction

Rubella is a highly contagious viral infection caused by rubella virus. Postnatally acquired rubella infection is generally mild and self-limiting in nature characterized by febrile illness with rash and lymphadenopathy [1]. However, mothers infected with rubella infection during early stage of pregnancy have 90% chance of passing the infection to the fetus [2,3]. Rubella infection just before conception or during the first trimester of pregnancy leads to miscarriage, fetal death, still birth or birth of an infant with congenital defects known as congenital rubella syndrome (CRS) [1]. Rubella is the leading vaccine-preventable cause of birth defects [4]. It has been estimated that in 2010 about 103,000 infants with CRS were born globally, and about half of them were from the South-East Asia Region (SEAR) [5].

Rubella vaccine is safe and efficacious and results in protective immunity in more than 90% of children vaccinated at or after one year of age [6]. Through large-scale vaccination programs, the Region of the Americas has already eliminated both measles and rubella, while WPRO, EMRO, AFRO and EURO have declared elimination targets [5]. In 2013, all countries of the WHO SEAR committed to eliminate measles and control rubella/CRS by 2020 by adopting four strategies: (a) achieving high coverage with two doses of measles and rubella containing vaccine (MRCV) in each district through routine and/or supplementary immunization activities (SIAs), (b) establishing and sustaining a case-based surveillance for measles and rubella/CRS, (c) developing and maintaining an accredited laboratory network and (d) developing linkages to other public health programs [5]. In 2019, the progress made towards measles elimination and control of rubella/CRS was reviewed and the WHO Regional Committee for SEA revised the target for elimination of measles and rubella by 2023 [7].

**Fig 1:**
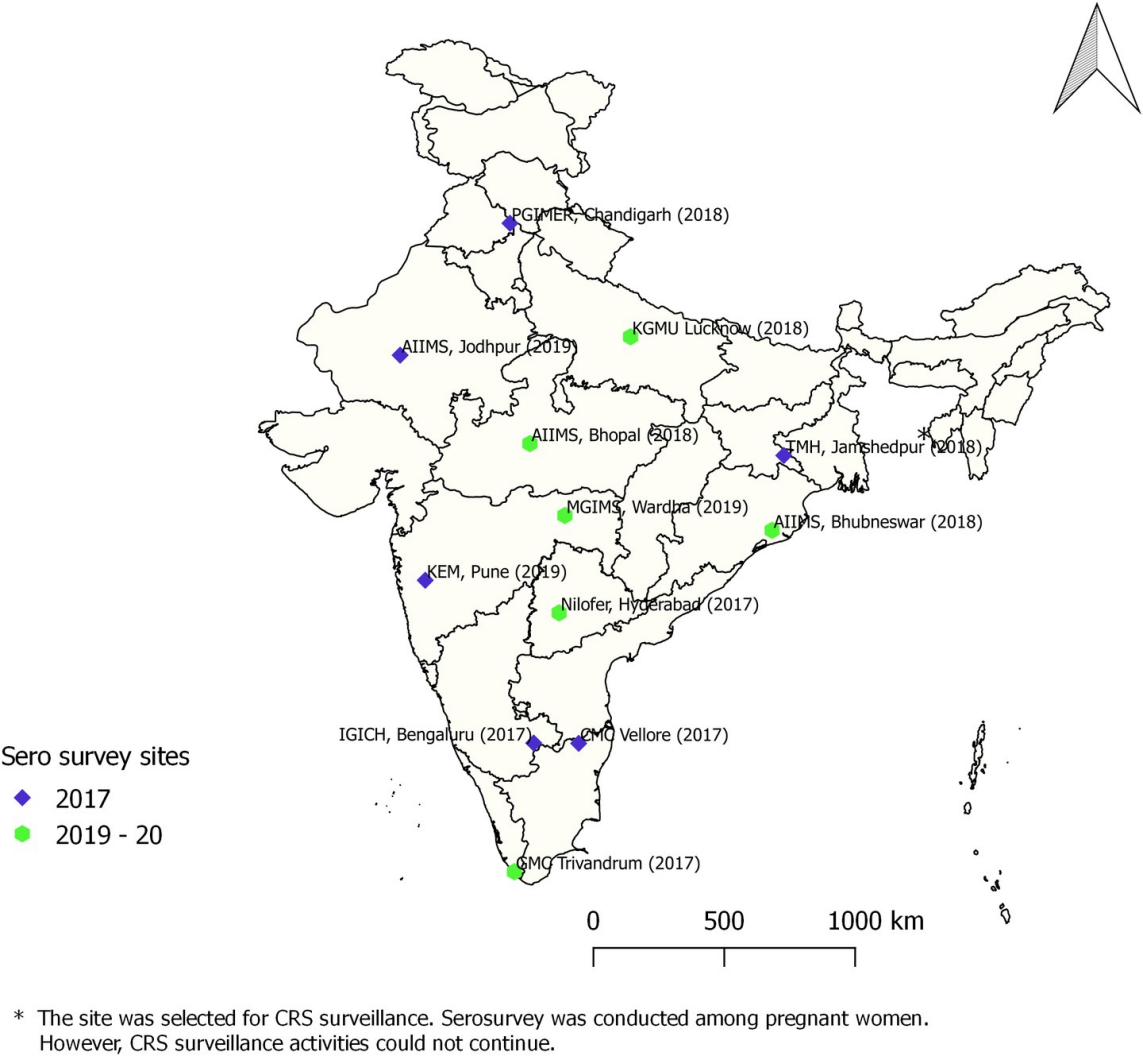
Location of CRS sentinel surveillance sites and year of MRCV SIA in India.

Towards achieving this goal, India conducted phased nationwide SIAs between 2017 and 2019, using MRCV targeting children aged 9 months to <15 years [8,9]. These SIAs have been completed in all Indian States, except Delhi and West Bengal, with high reported coverage [9,10]. Following these SIAs, MRCV was introduced in the routine childhood immunization, with the first dose given at the age of 9–12 months and second dose at the age of 16–24 months. Laboratory supported case-based surveillance for CRS was initiated in five sentinel sites (Phase-1) in 2016 with the objective of estimating disease burden of CRS and monitor its trend [11]. The ongoing surveillance was expanded in 2019 to include additional 6 sites (Phase-2) (S1 Fig). All the sentinel sites were located in states where MR-SIAs have been conducted (Fig 1). Analysis of surveillance data indicated that, of the 645 of clinically suspected cases enrolled during 2016–18 in five Phase-1 surveillance sites, 137 (21.2%) were classified as laboratory confirmed CRS [12].

As an adjunct to CRS surveillance, periodic serologic surveys were planned to monitor the rubella seroprevalence among the pregnant women over time. The serosurvey conducted in Phase-1 sites in 2017 indicated that 83.4% of the pregnant women had IgG antibodies against rubella [13]. This paper describes the rubella seroprevalence among pregnant women from six Phase-2 sentinel sites in 2019–20. Based on the seroprevalence data from the two phases, we estimated the incidence of CRS and the total number of CRS cases in India.

## Methods

### Ethics statement

The institutional human ethics committees of the ICMR-National Institute of Epidemiology, Chennai and ICMR-National Institute of Virology (NIV), Pune and the sentinel sites reviewed and approved the study. The site investigators explained the detailed study objectives to the participating pregnant women (or their legal guardians in case of minor) and a written informed consent was obtained. Only the consenting pregnant women were included in the study.

### Seroprevalence of IgG antibodies against rubella

The study was conducted among pregnant women attending antenatal clinics of the sentinel sites for the first-time during Oct 2019 –Jan 2020 (Fig 1). For an estimated seroprevalence of 80%, absolute precision of 5% and for 95% confidence interval, we required a sample size of 246 (rounded to 300) per site. We enrolled 300 pregnant women from each sentinel sites (totally 1800 samples from six sites) after obtaining written informed consent. Each site enrolled pregnant women consecutively, with a maximum of 10 women per day, in order to remove any chance of selection bias and to maintain quality of data. The enrolled women were interviewed to collect information about socio-demographic details, previous pregnancies and history of rubella vaccination. Two ml blood was collected from the women and sera were tested for IgG antibodies using commercial ELISA kit (Euroimmun, Luebeck, Germany). The antibody titers were expressed in international units per milliliter (IU/ml). Sera with anti-rubella IgG titres of <8 IU/ml were considered as seronegative, titers between 8 and 9.9 IU/ml as equivocal and titres of > = 10 IU/ml as seropositive for rubella. Women with antibody titer of > = 10 IU/ml were considered as seroprotected/immune, those with titer of <8 IU/ml as susceptible and those between 8 and 9.9 IU/ml as indeterminate susceptibility [14]. The assay was found to have high sensitivity (99.6%) and specificity (100%) as compared to other commercial test [15]. The survey methodology, in terms of sample size, sampling procedure, data collection and laboratory assay, was similar to the serosurvey conducted in 2017 [13].

For quality control (QC), 25% positive and 20% negative sera from each site were selected randomly and re-tested at the ICMR—NIV, Pune and exact titers of rubella IgG antibodies were estimated using the standard calibration sera corresponding to serum IgG titers of 1 IU/ml, 10 IU/ml, 50 IU/ml and 200 IU/ml, provided along with the Euroimmun kit.

The data were entered in a web-based portal and analyzed using STATA (Version 14) software to estimate the seroprevalence along with their 95% confidence interval (CI) (Wilson score CI) of anti-rubella IgG antibodies. The estimates were also presented by site and socio-demographic details. Chi-Square test and Fisher’s exact test were used to assess differences in seroprevalence in different categories. Statistical significance was set at p <0.05.

### CRS incidence and estimated number of CRS cases

We constructed catalytic models using R statistical software (version 3.6.2) to analyze the rubella antibody seroprevalence data from 2017 and 2019–2020 surveys from different sites. We constructed two models to fit the observed age-specific prevalence of susceptibility to rubella virus infection in study population using maximum likelihood method [16,17]. The constant force of infection model assumed that the force of infection (defined as the rate at which susceptible women are infected) was constant for all women of childbearing age, whereas the age varying force of infection model assumed that the force of infection varied with age (S1 Text). Since the number of women in the age group of 40–44 were few, we only considered the age-specific susceptibility data of women aged < = 39 years. The best fitting value for the force of infection observed was used to estimate the CRS incidence per 100,000 live births among women in reproductive age group in 5-year age interval between 15 and 39 years. The CRS incidence per 100,000 live births (I_CRS_(A)) by reproductive age group A (15–19, 20–24, 25–29, 30–34, and 35–39 years) was given by the following expression:

$$I_{CRS}(A)=S(A)(1-e^{-\lambda 16/52})*0.65*100,000$$

where S(A) is the proportion of susceptible women in age group A, λ is the constant force of infection estimated for the given population. Based on previous literature, the risk of child being born with CRS was assumed to be 65% if the mother was infected during the first 16 weeks of pregnancy and zero thereafter [17,18].

The CRS incidence among women aged between 15–39 years for a given year was calculated as the average CRS incidence per 100,000 live births for each 5-year age interval, weighted by the corresponding number of live births in women in each reproductive age group for a given year (t).


$$I_{CRS}(A_{15-39},\;t)=\frac{\sum_{i=1}^{5}I_{CRS}(A_{i})B_{e,i}(t)}{\sum_{i=1}^{5}B_{e,i}(t)}$$

Where A_i_ refers to age interval i, where i = 1,2, …, 5 corresponding to age interval 15–19, 20–24, 25–29, 30–34, and 35–39 years respectively. I_CRS_(A_i_) refers to CRS incidence corresponding to each age interval, B_e,i_ (t) refers to estimated number of live births in each age interval for a given year. The 95% CI for the constant and age specific force of infection and CRS incidence were obtained using non-parametric bootstrapping for binary data using quantile-based method obtained by 1000 bootstrap datasets associated with the serological dataset [19].

The number of live births was calculated by multiplying the age-specific fertility rates as per 2011 census data by the estimated number of women in each reproductive age interval in a given year. To obtain the number of births to rubella susceptible women in each age group, the estimated annual births among women in each age interval was multiplied by the proportion of women that were seronegative for rubella based on 2017 and 2019–20 serosurvey data in corresponding age interval. We estimated the number of infants born with CRS by multiplying the CRS incidence per 100,000 live birth with the estimated number of live births.

## Results

### Seroprevalence of IgG antibodies against rubella

We enrolled 1800 pregnant women from six sentinel sites across India. Majority of the women (n = 1385, 76.9%) were aged between 20 and 29 years, and 43.5% (n = 783) were from rural areas (Table 1). About half (n = 907, 50.4%) of them were primi-parous and 13.5% (n = 243) were in their first trimester of pregnancy.

**Table 1:** Seroprevalence of rubella among pregnant women from six surveillance sites, by selected socio-demographic characteristics, India, 2019–20.

Socio-demographic characteristics	No. tested	Positive		Negative		Indeterminate	
		No.	% (95% CI)	No.	% (95% CI)	No.	% (95% CI)
**Age group**							
16–19	87	69	79.3 (69.7, 86.5)	17	19.5 (12.6, 29.1)	1	1.1 (0.2, 6.2)
20–24	687	553	80.5 (77.4, 83.3)	128	18.6 (15.9, 21.7)	6	0.9 (0.4, 1.9)
25–29	698	586	84.0 (81.0, 86.5)	109	15.6 (13.1, 18.5)	3	0.4 (0.1, 1.3)
30–34	259	219	84.6 (79.7, 88.4)	39	15.1 (11.2, 19.9)	1	0.4 (0.1, 2.2)
35–45	69	54	78.3 (67.2, 86.4)	15	21.7 (13.6, 32.8)	0	-
**Education level**							
Illiterate	39	36	92.3 (79.7, 97.3)	3	7.7 (2.7, 20.3)	0	-
Up to 5th standard	79	68	86.1 (76.8, 92.0)	11	13.9 (8.0, 23.2)	0	-
6th to 10th standard	483	406	84.1 (80.5, 87.1)	73	15.1 (12.2, 18.6)	4	0.8 (0.3, 2.1)
11th standard to Graduate	1012	811	80.1 (77.6, 82.5)	194	19.2 (16.9, 21.7)	7	0.7 (0.3, 1.4)
Postgraduate	187	160	85.6 (79.8, 89.9)	27	14.4 (10.1, 20.2)	0	-
**Place of residence**							
Rural area	783	612	78.2 (75.1, 80.9)	165	21.1 (18.4, 24.1)	6	0.8 (0.4, 1.7)
Urban area	1017	869	85.4 (83.1, 87.5)	143	14.1 (12.1, 16.3)	5	0.5 (0.2, 1.1)
**Sentinel site**							
All India Institute of Medical Sciences, Bhubaneswar	300	243	81.0 (76.2, 85.0)	53	17.7 (13.8, 22.4)	4	1.3 (0.5, 3.4)
Niloufer Hospital, Hyderabad	300	266	88.7 (84.6, 91.8)	33	11.0 (7.9, 15.0)	1	0.3 (0.06, 1.9)
All India Institute of Medical Sciences, Bhopal	300	259	86.3 (82.0, 89.8)	40	13.3 (9.9, 17.6)	1	0.3 (0.06, 1.9)
Mahatma Gandhi Institute of Medical Sciences, Sewagram	300	240	80.0 (75.1, 84.1)	60	20.0 (15.9, 24.9)	0	-
King Georges Medical University, Lucknow	300	266	88.7 (84.6, 91.8)	34	11.3 (8.2, 15.4)	0	-
Govt Medical college, Trivandrum	300	207	69.0 (63.6, 74.0)	88	29.3 (24.5, 34.7)	5	1.7 (0.7, 3.8)
Overall	1800	1481	82.3 (80.4, 84.0)	308	17.1 (15.4, 18.9)	11	0.6 (0.3, 1.1)

Overall, IgG antibodies against rubella were found in 82.3% (n = 1481) women, 17.1% (n = 308) were seronegative, and 11 (0.6%) had indeterminate results. The rubella IgG seropositivity ranged between 69% (Trivandrum, Kerala) and 88.7% (Hyderabad, Telangana and Lucknow, Uttar Pradesh) in different sentinel sites. The seroprevalence was not different by age group and education level. Women residing in urban areas had higher seroprevalence as compared to those residing in rural areas (p<0.001) (Table 1).

### Estimation of rubella IgG antibody titers

As a part of QC, 432 sera were re-tested for estimation of rubella IgG antibody titers. The agreement between the IgG results at the sentinel sites and ICMR-NIV Pune was high (kappa = 0.92, p<0.001). The geometric mean titer of rubella antibodies among the seropositive women was 81.0 (95% CI: 71.2–92.1) IU/ml.

### CRS incidence and estimated number of CRS cases

We considered the seroprevalence data of 3585 women aged 16–39 years from 2017 and 2019–20 serosurveys. Of these, 2,970 (82.8%) were seropositive for IgG antibodies against rubella, 581 (16.2%) were seronegative and 34 (0.9%) were having indeterminate results (S1 Table). The seroprevalence was not different between the two surveys (p = 0.312). For building the catalytic models, women with indeterminate serology results were considered as seronegative.

**Fig 2:**
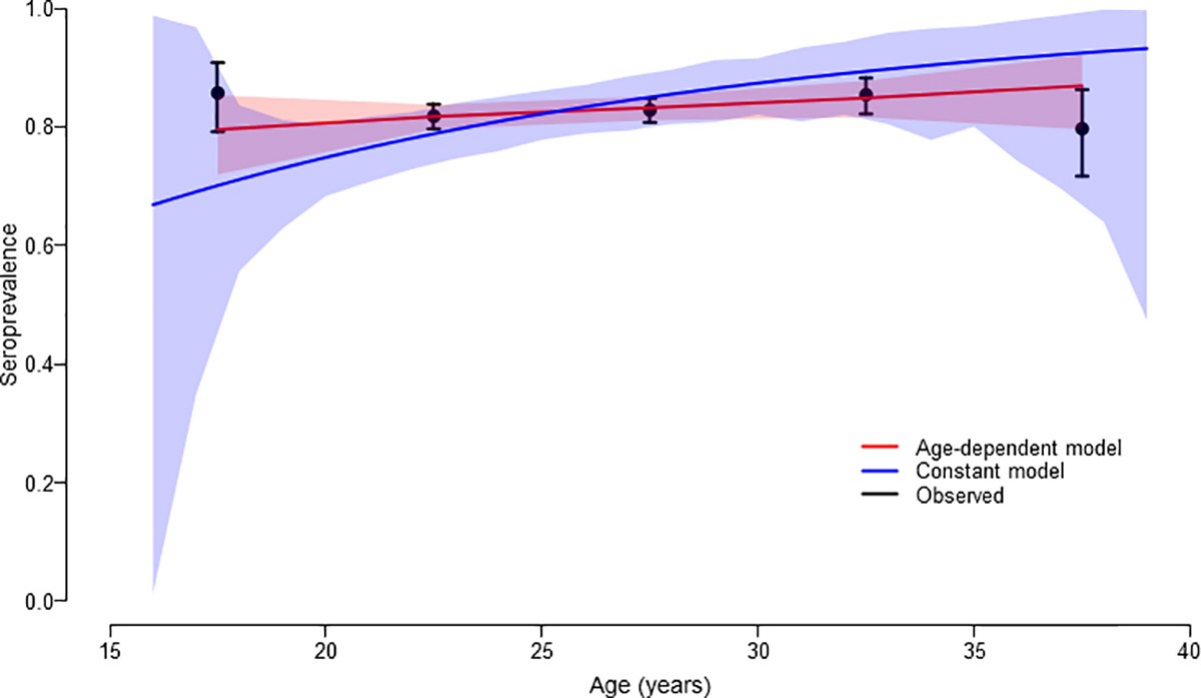
Observed and model-predicted seroprevalence of rubella by age (Data presented with 95% CIs). Base layer of the map can be found at http://www.naturalearthdata.com/about/terms-of-use/.

Fig 2 shows age-specific seroprevalence with fitted line and 95% CI based on constant as well as age-dependent force of infection models. With the constant force of infection model, the best fitting value for the force of infection was estimated to be 0.069 (95% CI: 0.066–0.072). The force of infection with the age-dependent model was estimated to be 0.030 (95% CI: 0.018–0.062) for women aged 16–19 years, 0.015 (95% CI: 0.009–0.029) for 20–24 years, 0.019 (95% CI: 0.009–0.038) for 25–29 years, 0.022 (95% CI: 0.011–0.056) for 30–34 years and 0.036 (95% CI: 0.020–0.096) for 35–39 years (Table 2). Based on these results, we estimated that 1 to 3 million women infected with rubella per year (Table 3). In 2019, approximately 22.18 million births were estimated in India from the estimated population of nearly 257.31 million women aged 16–39 years. Of these, 3,801,478 (17.1%) births would have been to rubella seronegative women. With the age-dependent force of infection model, it was estimated that 72,070 (95% CI: 50,685–101,218) pregnant women were infected with rubella every year and incidence of CRS was estimated to be 65.47 per 100,000 live births (95% CI: 41.60–104.16) in 2019 (Tables 2 and 3). The corresponding numbers for the constant force of infection model was 244,275 (95% CI: 235,506–252,087) pregnant women with rubella infection and incidence of CRS was 225.58 per 100,000 live births (95% CI: 217.49–232.41) (Tables 2 and 3). This incidence translates to an estimated 14,520 (95% CI: 9,225–23,100) and 50,028 (95% CI: 48,234–51,543) infants with CRS every year in India based on age-dependent and constant force of infection models respectively.

**Table 2:** Estimates of the force of infection based on constant and age-dependent catalytic models.

	Age-dependent force of infection			Constant force of infection		
Age group (in yrs)	Estimate (95% CI)	CRS incidence per 100,000 live births (95% CI)	Akaike information criterion	Estimate (95% CI)	CRS incidence per 100,000 live births (95% CI)	Akaike information criterion
16–19	0.03036 (0.01833, 0.06157)	65.47 (41.60, 104.16)	40.00821	0.06915 (0.06630, 0.07230)	225.58 (217.49, 232.41)	176.8444
20–24	0.01515 (0.00866, 0.02910)					
25–29	0.01901 (0.00892, 0.03838)					
30–34	0.02208 (0.01104, 0.05588)					
35–39	0.03621 (0.01996, 0.09598)					

**Table 3:** Estimated number of pregnant women aged 16–39 years infected with rubella infection in a year in India.

Age-group (years)	Estimated no. of women aged 16–39 years*	Fertility rate per 1000 women**	Annual expected births	Percentage of women who are rubella susceptible based on serosurvey data^$^	No. of births to rubella- susceptible women	Age-dependant force of infection model		Constant force of infection model	
						Estimated no. of women infected with rubella per year (95% CI)	Estimated no. of pregnant women infected with rubella per year (95% CI)	Estimated no. of women infected with rubella per year (95% CI)	Estimated no. of pregnant women infected with rubella per year (95% CI)
16–19	48712691	12.2	594295	14.29	84925	298262 (221515,495252)	3639 (2702,6042)	973314 (961826,982078)	11874 (11734, 11981)
20–24	59089941	122.9	7262154	18.20	1321712	162324 (119183,229985)	19950 (14648,28265)	866486 (844334,885461)	106491 (103769, 108823)
25–29	54952542	146.4	8045052	17.17	1381335	173676 (109265,237245)	25426 (15996,34733)	570270 (546996,591133)	83487 (80080, 86542)
30–34	48218732	94.7	4566314	14.59	666225	159422 (112750,237387)	15097 (10677,22481)	354122 (334355,372353)	33535 (31663, 35262)
35–39	46338709	36.9	1709898	20.31	347280	215667 (180505,262805)	7958 (6661,9698)	240838 (223837,256876)	8887 (8260, 9479)
Total	257312615		22177713	17.15	3801478	1009351 (743218,1462675)	72070 (50685,101218)	3005030 (2911348,3087901)	244275 (235506, 252087)

* 2019 Projected Population assuming 1.27% till 2015 and 1.07% from 2016 till 2019 (Ref: Population projection report 2019, Census of India)

** Sample Registration System (SRS) Statistical Report 2018

$ Based on 2017 and 2019–20 serosurveys

## Discussion

The findings of our serosurvey indicated that nearly one fifth of the pregnant women in six sentinel sites were susceptible to rubella. We estimated that the incidence of CRS in India was 65.5 per 100,000 live births with age-dependent force of infection model and 225.6 per 100,000 live births with constant force of infection model, translating into about 14,520–50,028 infants with CRS annually.

Govt of India is committed to eliminate measles and rubella by 2023. The facility-based surveillance for CRS revealed that about one-fifth of the suspected CRS patients during 2016–18 had evidence of laboratory confirmed rubella infection indicating continued transmission of rubella in India [12]. The nationwide SIAs conducted among children aged 9 months to <15 years using MR vaccine in 2017 is expected to reduce the transmission of rubella and thereby the burden of CRS in the country [8]. In order to monitor the progress made towards elimination of rubella in the country, it is essential to estimate the incidence of CRS [20]. Estimating the incidence of CRS based on hospital-based sentinel surveillance might not be accurate as these sentinel sites are tertiary hospitals catering to large population not only within the districts where these facilities are located but also neighboring districts as well states [11,12]. Hence, periodic serosurveys among antenatal women attending the sentinel sites have been included as an adjunct activity to facility-based CRS surveillance [13].

The serological survey conducted in six sentinel sites in India during 2019–20 indicated that more than 80% pregnant women were seropositive to rubella, while about 17% were susceptible to rubella infection. The proportion of women susceptible to rubella ranged between 11% and 29% in different sites. The earlier serosurvey conducted in 2017 in another six sites indicated seroprevalence of rubella infection was 83.4% (95% CI: 81.7–85.1) [13].

Compared to other states, the rubella seroprevalence was significantly lower in Kerala with more than one-fourth of the pregnant women susceptible for rubella. In India, majority of the population accesses immunization services through public sector. However, in high income states the private sector has been contributing substantially to the vaccine delivery [21]. A study based on the data on Indian private sector vaccine sales found that the private sector contributed to only 3.5% of measles vaccination in India during 2009–12. However, in the state of Kerala nearly 16% of children received measles containing vaccine from private sector [21]. Measles-Mumps-Rubella (MMR) vaccine has been available in the private sector for more than two decades and it is likely that children from urban areas of Kerala might have been receiving MMR vaccine. The rubella containing vaccine (RCV) however has not been in public sector till 2017 in most Indian state. The lower coverage of RCV is known to shift the age of rubella infection from childhood to the reproductive age [22], and thereby creating a pocket of low immunity.

The incidence of CRS in India based on the serological surveys conducted in 2017 and 2019–20 was 65.5 per 100,000 live births with age-dependent and 225.6 per 100,000 live births with constant force of infection models. The CRS incidence estimated based on simple catalytic (constant force of infection) model from earlier serosurveys conducted among women of reproductive age groups showed a wide variation in different sites, ranging between 87–262 per 100,000 live births [17,18]. Studies included in this analysis however were from 5 Indian cities (Delhi, Chandigarh, Lucknow, Calcutta and Vellore), with all studies except the one from Vellore conducted before 1990 using hemagglutination inhibition or Radial haemolysis assay. Seroprevalence observed in these studies ranged between 54–95%. Our estimate of CRS incidence was based on serosurveys conducted in 12 cities using the same sampling procedure and ELISA tests. The seroprevalence observed in our study was consistent with a recent review of published studies which reported that 12–30% of women in the reproductive age-group in India were susceptible to rubella infection [23]. Based on the estimated incidence of CRS, we estimated that every year 14,520–50,028 infants with CRS are born in India.

Our study has certain limitations. First, the health facilities for CRS surveillance were selected to represent different geographic regions and not randomly. Hence, the estimates of rubella seroprevalence might not be representative for the entire country. However, the baseline estimates of CRS incidence would be useful to monitor the trend of CRS incidence by conducting periodic serosurveys among antenatal mothers attending these sentinel surveillance sites. Second, the serosurveys were conducted at different time periods, first during a three-month period in 2017 and second during 2019–20. We used rubella susceptibility data from these surveys to develop the catalytic models. This however would not have affected our incidence estimates, as rubella seroprevalence would not have changed significantly over these three years as the nationwide MRCV- SIAs targeted children aged between 9 months to <15 years. Although the girls aged <15 years who had received MR vaccine during 2017 would be in the reproductive age group in 2019, only 1 woman in our survey was aged 16 years. Third, our serosurvey findings might not be representative of rural areas as 10 of the 12 sites where surveys were conducted were from urban areas and 68.9% and 56.5% women from the first and second surveys were from urban areas. Lastly, IgG antibodies against rubella may wane over time, and hence we might have under-estimated the rubella seroprevalence. Also, we did not evaluate the role of T cell mediated immunity against rubella.

In conclusion, the findings of our serosurvey indicated that about one fifth of women in the reproductive age group in India were susceptible for rubella. CRS continues to be an important public health problem in India, with an estimated incidence of 65.5 to 225.6 per 100,000 live births, translating into an estimated 14,520 to 50,028 infants with CRS born every year. The estimates of CRS incidence will serve as a baseline to monitor the impact of MRCVs conducted nationwide, as well progress towards the goal of rubella/CRS elimination in India, by periodically conducting follow-up serosurveys.

## Supporting information

S1 Fig CRS surveillance and rubella serosurvey timelines. (DOCX)

S1 Text Construction of catalytic model based on age-specific seroprevalence of rubella antibodies. (DOCX)

S1 Table Seroprevalence of rubella among pregnant women from twelve surveillance sites, by age group, India, 2017 and 2019–20. (DOCX)
