# Supplementary material for: Burden of congenital rubella syndrome (CRS) in India based on data from cross-sectional serosurveys, 2017 and 2019–20
Source: PLoS Negl Trop Dis. 2021 Jul 23;15(7):e0009608. doi: 10.1371/journal.pntd.0009608 (PMC8376255; doi:10.1371/journal.pntd.0009608)
Supplement: S1 Fig — (DOCX) [file pntd.0009608.s001.docx]

**S1 Fig:** CRS surveillance and rubella serosurvey timelines

CRS Surveillance initiated in 5^*^ sites

Surveillance Continue

Additional 6 sites added

Oct 2019 – Jan 2020 Rubella serosurvey conducted in additional 6 sites

**Year**

……

…

Rubella serosurvey conducted in 6 sites

**Phase I Phase 2**

**November**

**2016 2017 2018 2019 2020**

* CRS Surveillance initiated in 6 phase 1 sentinel sites and rubella serosurvey was conducted in all the 6 sites. However, one of the CRS surveillance site could not initiate CRS surveillance and hence dropped.
