# Supplementary material for: Burden of congenital rubella syndrome (CRS) in India based on data from cross-sectional serosurveys, 2017 and 2019–20
Source: PLoS Negl Trop Dis. 2021 Jul 23;15(7):e0009608. doi: 10.1371/journal.pntd.0009608 (PMC8376255; doi:10.1371/journal.pntd.0009608)
Supplement: S1 Text — (DOCX) [file pntd.0009608.s002.docx]

**S1 Text: Construction of catalytic model based on age-specific seroprevalence of rubella antibodies**

Force of infection is defined as the rate at which susceptible women are infected. We constructed two catalytic models assuming constant and age specific force of infection. The simple constant force of infection model predicts that the proportion of susceptible (seronegative) women declines with increasing age at a constant rate λ. The probability of a susceptible women in age group A is,

S(A) = e-^λA,^

where λ is the force o f infection. Then the probability of seropositive women in the age group A is given by 1-S(A). The binomial function to estimate λ of all women in the age group A is:

L(A) = (1-S(A)) ^P(A)^ * S(A) ^n(A)-P(A)^,

where P(A) is the number of seropositive women in age group A and n(A) is the number of women in the age group A.

Another model was also fitted to allow the force of infection to vary with age. For this we stratified women in the ages from 15-39 into 5 age groups of 5 years interval. Ie., (15-19, 20-24, 25-29, 30-34, 35-39). Now, the probability of a susceptible women in age group A is now changed to:

S(A) = $exp(-5 x \sum_{i=1}^{A} \lambda_{i}-2.5 x \lambda_{A+1})$

where *A* is the index of the age group of the women, ie., A=1 when the women belongs to age group of 15-19, A=2 when the women belongs to age group of 20-24 etc., and λ_i_ are the average force of infection of the corresponding age groups. The probability of observing a seropositive woman in age group A is 1 – S(A). Hence, the binomial likelihood function of all women in age group A is:

L(A) = (1-S(A)) ^P(A)^ * S(A) ^n(A)-P(A)^,

where P(A) is the number of seropositive women in age group A and n(A) is the total number of women in the age group A.

We estimated the force of infection parameters with corresponding 95% confidence interval using non-parametric bootstrapping technique. To compare model fit, we used Akaike information criterion (AIC) and the model with lowest AIC favouring good fit.
