# Supplementary material for: Burden of congenital rubella syndrome (CRS) in India based on data from cross-sectional serosurveys, 2017 and 2019–20
Source: PLoS Negl Trop Dis. 2021 Jul 23;15(7):e0009608. doi: 10.1371/journal.pntd.0009608 (PMC8376255; doi:10.1371/journal.pntd.0009608)
Supplement: S1 Table — (DOCX) [file pntd.0009608.s003.docx]

**S1 Table: Sero-prevalence of rubella among pregnant women from twelve surveillance sites, by age group, India, 2017 & 2019-20**

| **Age-group** | **Number**  **tested** | **Number**  **Positive** | **% (95% CI)** | **Number**  **Negative** | **% (95% CI)** | **Number**  **Indeterminate** | **% (95% CI)** |
| --- | --- | --- | --- | --- | --- | --- | --- |
| 16-19 | 154 | 132 | 85.7  (79.3, 90.4) | 21 | 13.6  (9.1, 19.9) | 1 | 0.6  (0.1-3.6) |
| 20–24 | 1368 | 1119 | 81.8  (79.7, 83.8) | 235 | 17.2  (15.3, 19.3) | 14 | 1.0  (0.6, 1.7) |
| 25–29 | 1380 | 1143 | 82.8  (80.7, 84.7) | 223 | 16.2  (14.3, 18.2) | 14 | 1.0  (0.6, 1.7) |
| 30–34 | 555 | 474 | 85.4  (82.2, 88.1) | 77 | 13.9  (11.2, 17.0) | 4 | 0.7  (0.3,1.8) |
| 35-39 | 128 | 102 | 79.7  (71.9, 85.7) | 25 | 19.5  (13.6, 27.2) | 1 | 0.8  (0.1, 4.3) |
| Overall | 3585 | 2970 | 82.8  (81.6, 84.0) | 581 | 16.2  (15.0-17.4) | 34 | 0.9  (0.7, 1.3) |
